# PmDNE: Prediction of miRNA-Disease Association Based on Network Embedding and Network Similarity Analysis

**DOI:** 10.1155/2020/6248686

**Published:** 2020-12-07

**Authors:** Junyi Li, Ying Liu, Zhongqing Zhang, Bo Liu, Yadong Wang

**Affiliations:** ^1^School of Computer Science and Technology, Harbin Institute of Technology (Shenzhen), Shenzhen, Guangdong 518055, China; ^2^School of Computer Science and Technology, Harbin Institute of Technology, Harbin, Heilongjiang 150001, China

## Abstract

Successful prediction of miRNA-disease association is nontrivial for the diagnosis and prognosis of genetic diseases. There are many methods to predict miRNA and disease, but biological data are numerous and complex, and they often exist in the form of network. How to accurately use the features of miRNA and disease-related biological networks to predict unknown association has always been a challenge. Here, we propose PmDNE, a method based on network embedding and network similarity analysis, to predict the miRNA-disease association. In PmDNE, the structure of network bipartite graph is improved, and a random walk generator is designed. For embedded vectors, 128 dimensions are used, and the accuracy of prediction is significantly improved. Compared with other network embedding methods, PmDNE is comparable and competitive with the state of art methods. Our method can solve the problem of feature extraction, reduce the dimension of features, and improve the efficiency of miRNA-disease association prediction. This method can also be extended to other area for biomedical network prediction.

## 1. Introduction

microRNA (miRNA) is a kind of noncoding RNA with length of around 22 nucleotides. It has been found in plants, animals, and viruses. Recent studies have shown that microRNAs play an important role in different biological processes [1]. It is able to prevent tumor invasion, control cell growth, regulate cell cycle regulation, and so on. Studies have also shown that many miRNAs are involved in human diseases [2], such as cancer, viral diseases, and immune-related diseases [3–5]. Therefore, successful prediction of disease-related miRNAs is nontrivial for the diagnosis and prognosis of genetic diseases and drug development.

How to predict human miRNA in the relationship and make good use of the existing miRNA disease association data is an important topic in the study of human diseases. For biomedicine, the accuracy of data is very important. There are many public databases related to miRNAs such as mir2disease [6], miRBase [7], and TarBase [8]. With the increasing concern of the scientific community on the relationship between diseases and miRNAs, their data are also included. For instance, HMDD [9] is the miRNA human disease association database established in 2007.

There are two kinds of major methods to predict disease-miRNA association. The first method is based on traditional network iteration, and the second one is based on machine learning.

In the traditional iterative method, the miRNAs and the nodes in the disease network are iterated, and the possible relationship is found from high to low through the final convergence result ranking. In 2016, Chen et al. suggested that global network similarity can capture the association between disease and miRNA more effectively than traditional local network similarity. Therefore, RWRMDA [10] method was developed to predict potential miRNA-disease associations. Chen et al. also proposed a computational model of HGIMDA [11], which integrates the known miRNA disease association, different types of disease similarity, and miRNA similarity into the heterograph to predict new disease-related miRNAs. However, the method of using network has its own disadvantages. It may be biased towards the well-known miRNAs and diseases. In the network method, restarting random walk is very time-consuming and parameters with transition probability; different selection of parameters also affects the final results. The experimental results of this kind of method highly depend on the reliable biological network model and cannot be applied to new miRNAs or new diseases.

The second kind of method is based on machine learning. This kind of method is able to solve the problem of new miRNAs and disease relation prediction. In 2011, Xu introduced a method [12] based on miRNA target imbalance network (MTDN) to give priority to new disease-related miRNAs. A weighted KNN-based HDMP [13] method is proposed by Xuan et al. In addition, the semantic similarity and phenotypic similarity of diseases are used to calculate the functional similarity matrix of miRNA. Chen proposed a semisupervised learning RLSMDA [14] model to predict potential disease-related miRNAs in 2014. RLSMDA [14] can calculate miRNA disease association prediction score of new diseases. This kind of method needs to solve two major problems: feature extraction and negative case missing.

Recently, people pay more and more attention to the network embedding method [15, 16]. It extracts features by extracting some relations of complex data and embeds the high latitude features of complex data into low dimensional space. In order to better predict the relationship between disease and miRNA, the network embedding method can be used to solve the problem of feature extraction. Therefore, we propose a method based on network embedding and network similarity analysis called PmDNE to predict the miRNA-disease association. In PmDNE, the structure of network bipartite graph is improved, and a random walk generator is designed. The accuracy of prediction has improved. Compared with other network embedding methods, PmDNE is comparable and competitive with the state of art methods. Our method can solve the problem of feature extraction, reduce the dimension of features, and improve the efficiency of miRNA-disease association prediction. This method can also be extended to other area for biomedical network prediction.

## 2. Materials and Methods

### 2.1. miRNA Disease Association Data

miRNA disease association data is obtained from the database HMDD3.0 (http://www.cuilab.cn/hmdde). In order to predict the effect effectively, we use the latest version of HMDD3.0. Some other databases, such as mri2disease, are not up to date. Some databases do not focus on the relationship between miRNA and disease, so we chose HMDD. A total of 894 disease nodes and 1208 miRNA nodes are obtained from the HMDD database, and 18733 diseases and miRNA association relationships are obtained as shown in Tables 1 and 2.

### 2.2. Disease Similarity Data about the Disease Similarity Network

We construct a directed acyclic graph (DAG) to describe the disease according to the literature [17] of Wang et al. Based on the medical subject title descriptor, it can be downloaded from the national medical library (http://www.nlm.nih.gov/). A total of 414003 related disease similarity relationships were obtained as shown in Table 2.

### 2.3. miRNA Similarity Data

miRNA similarity network is based on the method of calculating miRNA functional similarity proposed by Wang et al. [17]. The functional similarity of 495 miRNA nodes was obtained by downloading miRNA function similarity data conveniently.

### 2.4. Isomorphic Network Construction and Binetwork Construction

When constructing miRNA-disease binetwork, if there is correlation, the weight of their edges is 1, and the weight of nonexistent edges is 0. In this way, we can transform the prediction method into a binary classification problem. For isomorphic network, the weight of similarity data is set as the weight of isomorphic network.

### 2.5. The First Similarity Obtains the Node Embedding Vector of Graph Representation Learning

In order to better reconstruct the original network in the low dimensional space after embedding, the first similarity relation is represented by the existing edge learning, and the second similarity relationship is represented by the edge learning with transitive relationship. The final node representation is learned by combining the two methods. The modelling of explicit relationship is the same as the first similarity of Line [18]. By considering local similarity, the compactness of two connected nodes is defined as Equation (1).

(1) $$\begin{array}{c} \left(i,j\right)=\frac{W_{ij}}{\sum e_{ij}\in Ew_{ij}}, \end{array}$$

where *w*_*ij*_ is the edge *e*_*ij*_'s weight. The denominator is the sum of the weights of all edges. If two nodes are linked together, the probability of two nodes appearing together after embedding is very high.

Many research works [19, 20] have achieved good results on measuring the similarity of two nodes embedded in the space. Most of them refer to the idea of taking vector inner product of word2vec [21]. Herein, we also use this method to define the possibility of two nodes adjacent to each other in the embedded space as Equation (2).

(2) $$\begin{array}{c} \hat{\;P}\left(i,j\right)=\frac{1}{1+\exp\left(-{{\overset{⃑}{U}}_{i}}^{T}{\overset{⃑}{V}}_{j}\right)}, \end{array}$$

where *u*_*i*_*v*_*j*_ is the embedded node vector. Embedding vector means minimizing the difference between two nodes. That is, the closer the original node is, the closer the embedded node is still the closest relationship. To minimize the difference of the possibility of appearing together before and after embedding, KL divergence is used.

(3) $$\begin{array}{c} \mathrm{Minimize}\;O_{1}=\text{KL}\left(P\;|\;\left|\hat{P}\right.\right)=\underset{e_{ij}\in E}{\sum }P\left(i,j\right)\log\left(\frac{P\left(i,j\right)}{\hat{P}\left(I,J\right)}\right)=\propto -\underset{e_{ij}\in E}{\sum }w_{ij}\log\hat{P}\left(i,j\right). \end{array}$$

The equation above represents that the closer the distance between the two nodes before and after embedding, the smaller the KL divergence is, the more similar the two distributions are. The local information of the original network is retained through the first similarity relationship. In other words, for two closely connected nodes, the representation of the two nodes learned in this way is also close to each other in the low dimensional vector space.

### 2.6. The Second Similarity Obtains the Node Embedding Vector of Graph Representation Learning

We model the second similarity relationship and extract the feature vector by DeepWalk [20]. But the feature vectors embedded by the random walk of DeepWalk are all based on the same type of nodes. Therefore, we embed the nodes based on such a theory. Although there are no directly connected edges between two nodes of the same type, if there is a path from *u*_*i*_ to *u*_*j*_, it can be considered that there is a relationship between the two nodes. If two nodes of the same type are connected to the same node, they can be considered as having links. In PmDNE, we need to split a bipartite graph into two homogeneous networks, and combine it with miRNA network similarity and disease semantic similarity network. Through Equations (4) and (5), we generate two corpora containing different types of nodes. Then the random walk model is used to determine the node sequence library, and skipgram is used to obtain the similarity feature vector.

(4) $$\begin{array}{c} {W_{ij}}^{D}=\underset{k\in m}{\sum }w_{ik}w_{jk}+cd_{ij}, \end{array}$$

(5) $$\begin{array}{c} {W_{ij}}^{M}=\underset{k\in d}{\sum }w_{ik}w_{jk}+em_{ij}, \end{array}$$

where *w*_*ik*_*w*_*jk*_ is the weight from *i* to *j* and *j* to *k* and *d*_*ij*_ is the weight of nodes *i* to *j* in the disease similarity network. *m*_*ij*_ is the weight of nodes *i* to *j* in miRNA network. *c* and *e* are the weights of similarity networks.

However, the random walk strategy of DeepWalk is not optimal, so we redesign a random walk method. The specific way is as follows:

1. Obtain two networks with the same type of nodes by Equations (4) and (5) and construct two homogeneous networks by combining disease semantic similarity and miRNA functional similarity
2. The more links for one node, the more important the proof is, and the more random walk sequences start from it
3. Many random walk strategies [22] are to produce fixed length sequences, which does not conform to the actual rule of node embedding. The number of words in each sentence is uncertain. Therefore, we obtain node sequences of different lengths by making random walk stop or return to the original initial node at a certain step. The algorithm of measuring node importance we can chooses centrality algorithm or hits [23].  Pseudocode 1 shows the pseudocode of node sequence obtained by random walk.

Then, skipgram [24] algorithm is used to learn the embedded vector.

(6) $$\begin{array}{c} \mathrm{Maximize}\;O_{2}=\underset{u_{i}\in S\wedge S\in D^{U}}{\prod }\underset{u_{C}\in C_{s}\left(u_{i}\right)}{\prod }p\left(u_{c}\;|\;u_{i}\right), \end{array}$$

(7) $$\begin{array}{c} \mathrm{Maximize}\;O_{3}=\underset{v_{j}\in S\wedge S\in D^{v}}{\prod }\underset{v_{C}\in C_{s}\left(v_{j}\right)}{\prod }p\left(v_{c}\;|\;v_{j}\right), \end{array}$$

where *p*(*u*_*c*_ | *u*_*i*_) softmax is used for output.

(8) $$\begin{array}{c} p\left(u_{c}\;|\;u_{i}\right)=\frac{\exp\left({\overset{⃑}{u_{i}}}^{T}\overset{⃑}{\theta_{c}}\right)}{\sum_{k=1}^{\;|\;U\;|\;}\exp\left({\overset{⃑}{u_{i}}}^{T}\overset{⃑}{\theta_{k}}\right)}, \end{array}$$

(9) $$\begin{array}{c} p\left(v_{c}\;|\;v_{j}\right)=\frac{\exp\left({\overset{⃑}{v_{j}}}^{T}\overset{⃑}{\theta_{c}}\right)}{\sum_{k=1}^{\;|\;V\;|\;}\exp\left({\overset{⃑}{v_{j}}}^{T}\overset{⃑}{\theta_{k}}\right)}. \end{array}$$

However, due to the large amount of denominator calculation of softmax, we adopt the method of negative sampling [24–26], which transforms the calculation of each context vector into a binary classification problem of noncontext vector and context vector.

(10) $$\begin{array}{c} p\left(u_{c},N_{S}^{NS}\left(u_{i}\right)\;|\;u_{i}\right)=\underset{z\in u_{c}\wedge N_{S}^{NS}\left(u_{i}\right)}{\prod }p\left(z\;|\;u_{i}\right), \end{array}$$

(11) $$\begin{array}{c} p\left(z\;|\;u_{i}\right)=\left\{\begin{array}{cc} \sigma \left(\left({\overset{⃑}{u_{i}}}^{T}\overset{⃑}{\theta_{z}}\right)\right) & \text{if }z\text{ is }u_{i}^{\prime }s\text{ context}\\ 1-\sigma \left({\overset{⃑}{u_{i}}}^{T}\overset{⃑}{\theta_{c}}\right) & z\in N_{S}^{NS}\left(u_{i}\right) \end{array}\right.. \end{array}$$

### 2.7. Obtain the Node Embedding Vector of the Final Graph Representation Learning

The function formula of the final optimization is Equation (12).

(12) $$\begin{array}{c} \mathrm{Maximize}\;L=\alpha \log O_{2}+\beta \log O_{3}-\gamma O_{1}. \end{array}$$

In the end, the embedding vector is obtained by iterating the embedding vector with random gradient descent [27]. For example, we use random gradient descent to update $\overset{⃑}{u_{i}}$ and $\overset{⃑}{v_{j}}$ for *O*_1_:

(13) $$\begin{array}{c} \overset{⃑}{u_{i}}=\overset{⃑}{u_{i}}+\lambda \left\{{\gamma w}_{ij}\left[1-\sigma \left({\overset{⃑}{u_{i}}}^{T}\overset{⃑}{v_{j}}\right)\right]*\overset{⃑}{v_{j}}\right\}, \end{array}$$

(14) $$\begin{array}{c} \overset{⃑}{v_{j}}=\overset{⃑}{v_{j}}+\lambda \left\{{\gamma w}_{ij}\left[1-\sigma \left({\overset{⃑}{u_{i}}}^{T}\overset{⃑}{v_{j}}\right)\right]*\overset{⃑}{u_{i}}\right\}, \end{array}$$

where *σ* is the sigmoid function and *λ* is the learning rate.

For *O*_2_ and *O*_3_, gradient descent is also used to update $\overset{⃑}{u_{i}}$ and $\overset{⃑}{v_{j}}:$

(15) $$\begin{array}{c} \overset{⃑}{u_{i}}=\overset{⃑}{u_{i}}+\lambda \left\{\underset{z\in \left\{u_{c}\right\}\cup N_{S}^{NS}\left(u_{i}\right)}{\sum }\alpha \left[I\left(z,u_{i}\right)-\sigma \left({\overset{⃑}{u_{i}}}^{T}\overset{⃑}{\theta_{z}}\right)\right]*\overset{⃑}{\theta_{z}}\right\}, \end{array}$$

(16) $$\begin{array}{c} \overset{⃑}{v_{j}}=\overset{⃑}{v_{j}}+\lambda \left\{\underset{z\in \left\{v_{c}\right\}\cup N_{S}^{NS}\left(v_{j}\right)}{\sum }\beta \left[I\left(z,v_{j}\right)-\sigma \left({\overset{⃑}{v_{j}}}^{T}\overset{⃑}{\vartheta_{z}}\right)\right]*\overset{⃑}{\vartheta_{z}}\right\}, \end{array}$$

where *I*(*z*, *u*_*i*_) is *i* in *u*_*i*_'s context, if exist *I*(*z*, *u*_*i*_) is 1 and 0 if not. *I*(*z*, *v*_*j*_) is similarity. For the centre word's contextual and noncontext word vectors $\overset{⃑}{\theta_{z}}$ and $\overset{⃑}{\vartheta_{z}}$, they are defined as (17) and (18).

(17) $$\begin{array}{c} \overset{⃑}{\theta_{z}}=\overset{⃑}{\theta_{z}}+\lambda \left\{\alpha \left[I\left(z,u_{i}\right)-\sigma \left({\overset{⃑}{u_{i}}}^{T}\overset{⃑}{\theta_{z}}\right)\right]*\overset{⃑}{u_{i}}\right\}, \end{array}$$

(18) $$\begin{array}{c} \;\overset{⃑}{\vartheta_{z}}=\overset{⃑}{\vartheta_{z}}+\lambda \left\{\beta \left[I\left(z,v_{j}\right)-\sigma \left({\overset{⃑}{v_{j}}}^{T}\overset{⃑}{\vartheta_{z}}\right)\right]*\overset{⃑}{v_{j}}\right\}. \end{array}$$

By considering both the first similarity relation and the second similarity relation, the node representation is learned. Then, we can use random forests to make predictions.

### 2.8. Criteria for Validation of Prediction

For the binary classification problem, according to the combination of real class and learner prediction category, the examples can be divided into true positive example (TP), false negative example (FN), false positive example (FP), and true negative example (TN) as shown in Table 3.

AUC is the area under the curve, and its calculation method takes into account the classification ability of the classifier for positive and negative cases. In the case of unbalanced samples, the classifier can still make a reasonable evaluation. The larger the AUC, the more advanced the prediction results of the samples and the better the prediction effect. In addition to the above two important indicators, we also select precision and accuracy; F1 scores and recall were used as the evaluation criteria.

## 3. Results and Discussion

### 3.1. Results

The 4 : 1 data set is divided into training set and test set, and the final features are obtained by five cross validation. The feature dimension is 128 dimensions, and 2102 node vectors are obtained. Because this is an unbalanced classification task, we solve this problem by randomly selecting the same number of unconnected edges as negative examples. The random forest [28] is used to predict the parameters. For the weight of the similarity between the two networks, we choose 0.5 that is half. The maximum number of steps max_t of random walk is 32, and the minimum number of steps is 1, 0.15 for the probability of stopping immediately. 0.0001, 0.01, and 0.1 are for the three optimization objective functions, respectively. The AUC values of ROC and PR are 0.8954 ± 0.001 and 0.9002 ± 0.0015.

We also measure the results of adding network similarity and not adding network similarity. The results shown in Table 4 are as follows: 1 is the embedding method with two similar networks added, 2 is the embedding method without adding network, 3 is the embedding method with adding disease network, and 4 is the result of adding miRNA similarity network. From the results, we can see the result of adding similar network. It is the best. This shows that we have greatly improved the prediction effect by adding network similarity.

### 3.2. Computational Efficiency

Because this paper uses Python implementation, so the time efficiency will be lower than other embedding methods completed by C++. C++ is closer to the bottom, so the efficiency will be improved. However, Python has many data processing-related libraries, which will make the code writing more convenient. In this paper, the running time efficiency is minute level, and other methods are seconds' level.

### 3.3. Parameter Analysis

**Figure 1:**
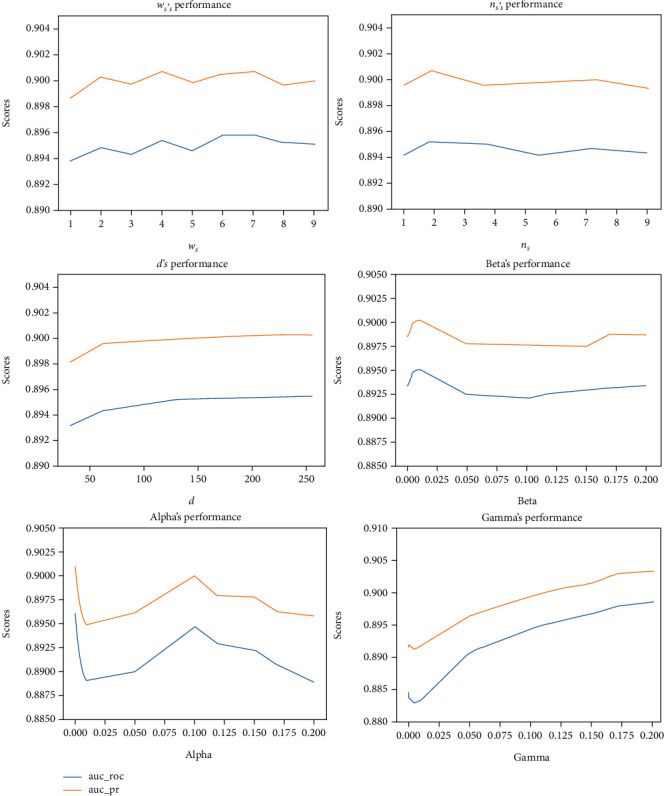
Influence of parameters on prediction effect. The parameter scores mean the value obtained by ROC or PR. The scores of alpha, beta, and gamma fluctuate greatly. These three parameters play an important role in regulating the size of the first similarity and the second similarity.

Important parameters are analyzed as shown in Figure 1. The parameter scores mean the value obtained by ROC or PR, and *w*_*s*_ is the size of the context window after selecting a central word in the random walk corpus. As the window becomes larger, the AUC of ROC and AUC of PR increase, which is in line with the actual law. The more context is, the more accurate the prediction will be. However, when the window reaches a certain value, AUC of ROC and AUC of PR tend to be stable, because the context information is enough to produce prediction results. *n*_*s*_ is the number of negative samples selected. With the increase of the number of negative samples, the prediction will be more accurate. *d* is the dimension of the embedded vector. It can be concluded that the higher the dimension is, the more original information it retains, and the more accurate the prediction is. But when it reaches a certain value, it also tends to be stable. *α*, *β*, and *γ* are the coefficients of the optimization function, respectively. It can be seen that the fluctuation is very obvious, which indicates that they are important parameters to balance the first similarity and the second similarity for the embedded vector. Single increase of explicit or implicit relationship will lead to the uneven proportion of the first similarity and the second similarity, which will lead to the fluctuation of the prediction results.

### 3.4. Comparison of Network Embedding Methods

In order to compare the characteristics of this paper, we select six methods, such as DeepWalk, line, node2vec, grarep [29], GF, lap [30], and LLE [31], and we compare the results. The same data and prediction methods are used to measure the performance of this method. The following are the introduction of some of these methods and the results of comparative experiments.

DeepWalk: a node embedding method for heterogeneous networks, which obtains node sequences through unbalanced random walks, and then uses word2vec to obtain embedding vectors

Line: by optimizing the first and second similarity in a heterogeneous network, the final node vector is obtained

Node2vec: inherits DeepWalk and generates node sequence through organized random walk

Grarep: using matrix decomposition to solve network embedding problem. It can deal with weighted networks and integrate the global structure information in the process of learning network representation. However, due to the large amount of computation, this method will be particularly time-consuming, so it cannot be used in large-scale networks

GF: higher order nearest neighbor keeps embedding. By introducing higher order similarity matrix, higher-order similarity is preserved by generalized singular value decomposition to obtain embedding vector

**Figure 2:**
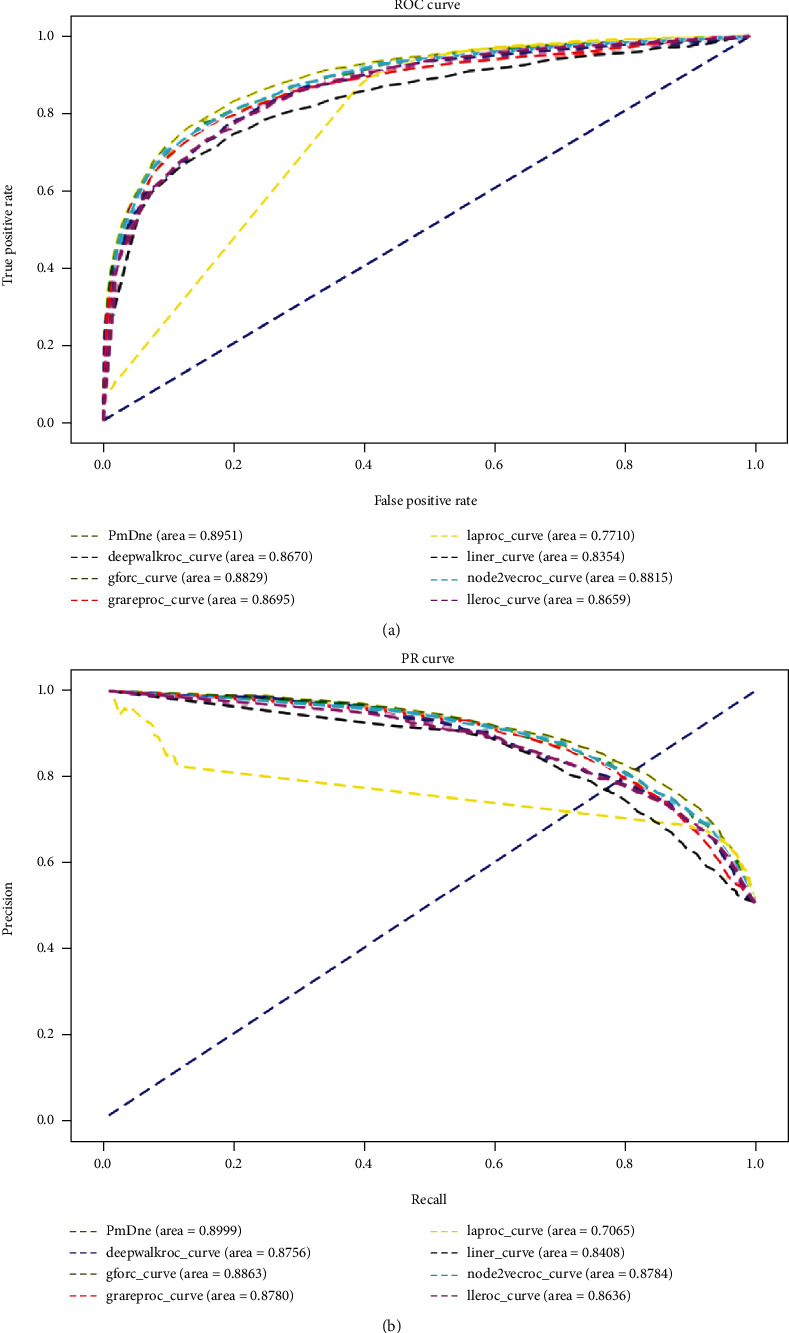
Experimental results for PR and ROC curves of each models: (a) ROC curves for all models; (b) PR curves for all models.

From Table 5 to Figure 2, we can see that the ROC and AUC of PR method in this paper are better than other network embedding methods.

### 3.5. Comparison of Different Classifiers

Table 6 shows the comparison of the prediction results of different classifiers on the embedded vector. We compared six classifiers. The RF is the Random Forest Classifier; KNN is the K Neighbors Classifier; ADBC is the AdaBoost Classifier; LR is the Logistic Regression Classifier; GBC is the Gradient Boosting Classifier; SVM is the support vector machines.

## 4. Conclusion

We propose PmDNE, a method based on network embedding and network similarity analysis, to predict the miRNA-disease association. For embedded vectors, 128 dimensions are used, and the accuracy of prediction is significantly improved. The values of PR and AUC of PmDNE are 0.9002 and 0.8954, respectively. Compared with other network embedding methods, PmDNE has better ability on extract the features of disease and miRNA networks. Our method improves the efficiency of miRNA-disease association prediction. This method can also be extended to other area for biomedical network prediction.

## Figures and Tables

**Pseudocode 1:**
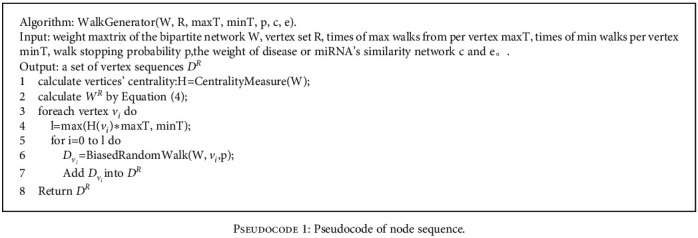
Pseudocode of node sequence.

**Table 1:** Number of edges about miRNA and disease, miRNA and miRNA, and disease and disease.

	miRNA	Disease
miRNA	644918	18733
Disease	18733	414003

**Table 2:** The number of miRNA nodes and disease node.

	Nodes number
miRNA	1208
Disease	894

**Table 3:** Concept of TF, FN, FP, and TN.

Prediction values	Actual values	
	Positive	Negative
Positive	TP	FN
Negative	FP	TN

PR curve: abscissa is recall rate and ordinate is precision; precision = TP/(TP + FP); recall = TP/(total positive samples) = TP/(TP + FN); ROC curve: the abscissa is FPR and the ordinate is TPR; TPR = TP/(TP + FN); FPR = FP/(TN + FP).

**Table 4:** Influence of different networks on results.

	ROC_AUC	PR_AUC	PREC	ACC	F1	Recall
1	0.8952 ± 0.003	0.9002 ± 0.002	0.6744 ± 0.01	0.8153 ± 0.02	0.8104 ± 0.004	0.7863 ± 0.004
2	0.8833 ± 0.002	0.8916 ± 0.0015	0.6480 ± 0.015	0.8034 ± 0.02	0.7986 ± 0.004	0.7861 ± 0.005
3	0.8906 ± 0.0015	0.8966 ± 0.002	0.663 ± 0.001	0.8103 ± 0.015	0.8054 ± 0.003	0.7857 ± 0.004
4	0.8914 ± 0.001	0.8968 ± 0.0015	0.6634 ± 0.003	0.8115 ± 0.02	0.8056 ± 0.002	0.7813 ± 0.004

**Table 5:** Comparison of network embedding methods.

	Auc_roc	Auc_pr
PmDNE	0.8954 ± 0.003	0.9002 ± 0.002
DeepWalk	0.8689 ± 0.002	0.8780 ± 0.002
Line	0.8302 ± 0.003	0.8305 ± 0.002
Node2Vec	0.8807 ± 0.004	0.8782 ± 0.004
GraRep	0.8766 ± 0.002	0.8760 ± 0.003
GF	0.8881 ± 0.004	0.8856 ± 0.003
Lap	0.7706 ± 0.004	0.7062 ± 0.002
lle	0.8670 ± 0.004	0.8673 ± 0.004

**Table 6:** Comparison of the effect of different classifiers.

	ROC_AUC	PR_AUC	PREC	ACC	F1	Recall
RF	0.8954 ± 0.003	0.9002 ± 0.002	0.6744 ± 0.01	0.8153 ± 0.02	0.8104 ± 0.004	0.7863 ± 0.004
KNN	0.8746 ± 0.002	0.8560 ± 0.0015	0.7933 ± 0.015	0.8075 ± 0.02	0.8014 ± 0.004	0.7758 ± 0.005
GBC	0.8827 ± 0.0015	0.8955 ± 0.002	0.7747 ± 0.001	0.8045 ± 0.015	0.7937 ± 0.003	0.7532 ± 0.004
SVM	0.7693 ± 0.001	0.8194 ± 0.0015	0.7367 ± 0.003	0.7042 ± 0.02	0.5989 ± 0.002	0.4495 ± 0.004
LR	0.8070 ± 0.02	0.8412 ± 0.005	0.7541 ± 0.004	0.7390 ± 0.015	0.7153 ± 0.004	0.65618 ± 0.005
ADBC	0.8330 ± 0.002	0.8579 ± 0.002	0.71370.0015	0.7570 ± 0.002	0.7348 ± 0.004	0.6757 ± 0.005

## Data Availability

miRNA disease association data is obtained from the database website: HMDD3.0, link: http://www.cuilab.cn/hmdde. Disease theme data is downloaded from the national medical library, link: http://www.nlm.nih.gov. The functional similarity of 495 miRNA nodes was obtained by downloading miRNA function similarity data [17].

## Supplementary Materials

Supplementary Materials Supplementary file 1: main code for PmDNA.
